# Experimental and Meta-Analytic Validation of RNA Sequencing Signatures for Predicting Status of Microsatellite Instability

**DOI:** 10.3389/fmolb.2021.737821

**Published:** 2021-11-23

**Authors:** Maksim Sorokin, Elizaveta Rabushko, Victor Efimov, Elena Poddubskaya, Marina Sekacheva, Alexander Simonov, Daniil Nikitin, Aleksey Drobyshev, Maria Suntsova, Anton Buzdin

**Affiliations:** ^1^ Laboratory For Clinical and Genomic Bioinformatics, I.M. Sechenov First Moscow State Medical University, Moscow, Russia; ^2^ Moscow Institute of Physics and Technology, Dolgoprudny, Russia; ^3^ OmicsWay Corp., Walnut, CA, United States; ^4^ Faculty of Biology, Lomonosov Moscow State University, Moscow, Russia; ^5^ World-Class Research Center “Digital Biodesign and Personalized Healthcare”, Sechenov First Moscow State Medical University, Moscow, Russia; ^6^ Oncobox Ltd., Moscow, Russia; ^7^ Shemyakin-Ovchinnikov Institute of Bioorganic Chemistry, Moscow, Russia

**Keywords:** microsatellite instability, RNA sequencing, NGS, RNAseq, gene signatures, experimental validation

## Abstract

Microsatellite instability (MSI) is an important diagnostic and prognostic cancer biomarker. In colorectal, cervical, ovarian, and gastric cancers, it can guide the prescription of chemotherapy and immunotherapy. In laboratory diagnostics of susceptible tumors, MSI is routinely detected by the size of marker polymerase chain reaction products encompassing frequent microsatellite expansion regions. Alternatively, MSI status is screened indirectly by immunohistochemical interrogation of microsatellite binding proteins. RNA sequencing (RNAseq) profiling is an emerging source of data for a wide spectrum of cancer biomarkers. Recently, three RNAseq-based gene signatures were deduced for establishing MSI status in tumor samples. They had 25, 15, and 14 gene products with only one common gene. However, they were developed and tested on the incomplete literature of The Cancer Genome Atlas (TCGA) sampling and never validated experimentally on independent RNAseq samples. In this study, we, for the first time, systematically validated these three RNAseq MSI signatures on the literature colorectal cancer (CRC) (*n* = 619), endometrial carcinoma (*n* = 533), gastric cancer (*n* = 380), uterine carcinosarcoma (*n* = 55), and esophageal cancer (*n* = 83) samples and on the set of experimental CRC RNAseq samples (*n* = 23) for tumors with known MSI status. We found that all three signatures performed well with area under the curve (AUC) ranges of 0.94–1 for the experimental CRCs and 0.94–1 for the TCGA CRC, esophageal cancer, and uterine carcinosarcoma samples. However, for the TCGA endometrial carcinoma and gastric cancer samples, only two signatures were effective with AUC 0.91–0.97, whereas the third signature showed a significantly lower AUC of 0.69–0.88. Software for calculating these MSI signatures using RNAseq data is included.

## Introduction

Microsatellite instability (MSI) results from and is a marker of defective DNA mismatch repair (dMMR). Tumors accumulate multiple mutations across the genome (Ryan et al., 2017). Short tandem repeats are particularly frequent targets to mismatch errors, and dMMR-linked mutations are prone to be present in microsatellite regions (tandem repeats of up to six nucleotides short stretches of DNA) (Johansen et al., 2019). Detectable expansion or shrinkage of microsatellite repeats is referred to as MSI (Marcus et al., 2019).

MSI was the second clinically approved predictive biomarker for the PD1-specific immunotherapy in adult and pediatric advanced cancer patients. In 2017, the approval of the PD1-specific checkpoint inhibitor antibody pembrolizumab for patients with high MSI was based on the evidence of clinical efficacy from five clinical trials (Marcus et al., 2019). This was the first time when a cancer drug was approved based on a general, not a tumor type-specific biomarker.

Tumors with dMMR also have more mutations in non-microsatellite DNA and thus have more neoantigens. For example, an average figure of ∼1,800 mutations and ∼580 neoantigens was detected in colorectal cancers (CRCs) with dMMR compared with only ∼70 mutations and ∼20 predicted neoantigens in CRCs with normal MMR (Le et al., 2015). An increased amount of neoantigens in dMMR tumors promotes tumor infiltration by lymphocytes (Dudley et al., 2016; Giannakis et al., 2016), which may cause a more effective response to immunotherapy (Luchini et al., 2019). This provides a theoretical basis for MSI/dMMR biomarker effectiveness for the treatment response to immune checkpoint inhibitors targeting PD-1, PD-L1, and CTLA-4 proteins (Le et al., 2015).

The Food and Drug Administration did not specify which assay should be used to measure MSI. Currently, there are three basic options available for determining MSI status in clinical practice: immunohistochemistry (IHC) for testing dMMR, polymerase chain reaction (PCR), and genomic/exome/panel sequencing for detecting MSI (Ryan et al., 2017; Baretti and Le, 2018; Waalkes et al., 2018).

IHC test interrogates expressions of four proteins: MLH1, MSH2, MSH6, and PMS2. dMMR is diagnosed when there is detected loss of expression of one or more such proteins (Danaher et al., 2019). IHC tests for dMMR/MSI is simple and cost-effective, but it has a downside of relatively low analytic accuracy due to technical inconsistencies such as tissue fixation issues (Engel and Moore, 2011) and biological reasons such as missense mutations in MMR genes that can functionally inactivate protein without altering its IHC-tested expression level (Shia, 2008).

Alternatively, several PCR MSI panels have been designed, and two are most frequently used in practice: (1) two mononucleotide (*BAT-25* and *BAT-26*) and three dinucleotide (*D5S346*, *D2S123*, and *D17S250*) repeat panel (Boland et al., 1998) and (2) five poly-A mononucleotide (*BAT-25*, *BAT-26*, *NR-21*, *NR-24*, and *NR-27*) repeat panel. The latter has greater sensitivity and specificity compared with the (1) panel (Suraweera et al., 2002). Moreover, unlike (1), panel (2) has no requirement of having both tumors and paired healthy tissue for the test (Shemirani et al., 2011). If at least two biomarkers in either panel lose stability, the tumor is diagnosed as MSI-positive.

As PCR testing is based on a limited number of specific microsatellite sites, this approach cannot capture full microsatellite profiles and thus cannot detect ∼0.3–10% of MSI cases (16). Furthermore, MSI prevalence and type are markedly different across the different cancer types. For example, lung, breast, and prostate cancers have only ∼1–2% MSI incidence (Luchini et al., 2019; Marcus et al., 2019). This proportion is higher for gastric, ovarian, and cervical cancers and is maximal for CRC. These observations are reflected in specific diagnostic guidelines, and MSI testing is not routinely recommended for most tumor types. These factors limit the use of the PCR MSI test on a broad scale (Wang et al., 2021).

DNA sequencing tests use either whole-exome sequencing (WES) or cancer gene panels. For targeted gene panels, the number of genes varies from around 200 to >5,000 genes (Waalkes et al., 2018). Thus, the analytic sites for testing MSI are strongly different among the different targeted panels, whereas the WES approach can provide more objective data, as evidenced by ∼100% agreement with gold standard IHC and PCR MSI testing methods for 130 CRC patients when using the MSI sensor method (Johansen et al., 2019).

As opposed to IHC- or PCR-based MSI testing, which are most suitable for CRC and other cancers belonging to the spectrum of Lynch syndrome, the sequencing MSI approach can be used for more tumor types. It can provide an advantage of combining MSI analysis with mutation screening and tumor mutation burden analysis (Wang et al., 2021). However, genomic deep sequencing-based testing has major challenges of high cost and lack of wide availability (Waalkes et al., 2018).

**FIGURE 1:**
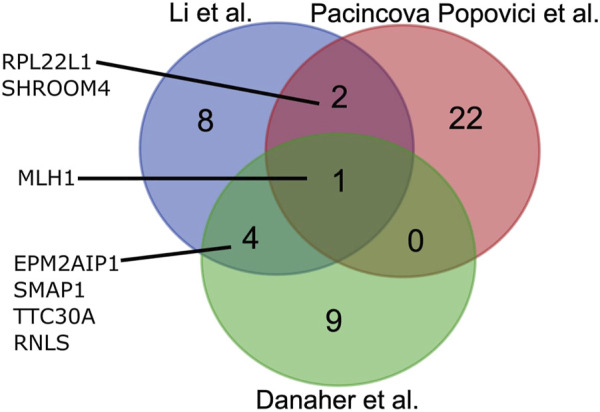
Overlap between gene composition of MSI expression signatures developed by Li et al. (2020), Pačínková and Popovici (2019), and Danaher et al. (2019).

On the other hand, RNA sequencing (RNAseq) can provide another type of data for MSI assessment. In turn, the RNAseq approach has several serious advantages that make it another candidate for an emerging method of choice for MSI testing. RNAseq is a well-established technology for tumor specimens, including formalin-fixed, paraffin-embedded (FFPE) tissue samples (Buzdin et al., 2020). Typically, one RNAseq analysis is less expensive than for WES or panel genomic sequencing (Bossel Ben-Moshe et al., 2018). It can be informative for the assessment of IHC biomarkers (Sorokin et al., 2020c; 2020b), expression of cancer drug target genes (Buzdin et al., 2020; Sorokin et al., 2020d), tumor-specific molecular pathway activation (Buzdin et al., 2018; Borisov et al., 2020a), for personalized modeling of tumor drug response (Kim et al., 2020; Tkachev et al., 2020), and even for tumor mutation burden assessment (DiGuardo et al., 2021). Furthermore, RNAseq data that inform on total gene expression profiles can also be applicable for generating MSI gene signatures. Three such signatures were recently developed (Danaher et al., 2019; Pačínková and Popovici, 2019; Li et al., 2020) based on TCGA project (Tomczak et al., 2015) publicly available RNAseq data for CRC samples annotated with MSI status by gold standard IHC and/or PCR methods. A signature established by Li et al. (2020) includes 25 genes, a signature by Pačínková and Popovici (2019) includes 15 genes, and a double signature by Danaher et al. (2019)—14 genes. Interestingly, those signatures are mostly different by gene content and have only one common gene (Figure 1).

However, these signatures were developed and validated on the same TCGA samplings and were never validated experimentally on independent RNAseq profiles. In this study, we, for the first time, systematically validated these three RNAseq MSI signatures on the literature CRC (*n* = 619), endometrial carcinoma (*n* = 533), gastric cancer (*n* = 380), uterine carcinosarcoma (*n* = 55), and esophageal cancer (*n* = 83) samples and on the set of experimental CRC RNAseq samples (*n* = 23) for the tumors with known MSI status. As the gold experimental standard, we used seven PCR MSI biomarkers.

We found that all three signatures performed well with area under the curve (AUC) ranges of 0.94–1 for the experimental CRCs and 0.94–1 for the TCGA CRC, esophageal cancer, and uterine carcinosarcoma samples. However, for the TCGA endometrial carcinoma and gastric cancer samples, only two signatures were effective with AUC 0.91–0.97, whereas the third signature showed a significantly lower AUC of 0.69–0.88. Finally, we provide software for calculating these MSI signatures using RNAseq data.

## Results

### Microsatellite Instability Data Curation and Analysis

For the literature (TCGA) dataset, we extracted MSI statuses for 1,670 available RNAseq samples from the Broad Firehose webpage. These MSI statuses obtained using IHC or PCR profiling were then considered as the gold standards for the assessment of transcriptomic signatures. As only MSI-high tumors are considered for specific therapeutic options, we pooled MSI-low and MSS (microsatellite stable) samples in a single class for further analyses. Totally, we obtained 1,340 MSI-low/MSS and 330 MSI-high profiles. These samples represented CRC, endometrial carcinoma, gastric cancer, uterine cancer, and esophageal cancer (Table 1). This was higher than the samplings used previously to validate *Li*, *Pacinkova and Popovici*, and *Danaher* signatures in the original studies (a total of 1,302, 626, and 689 samples, respectively; Table 1). We checked RNAseq gene signatures in binary classifier mode.

**TABLE 1:** Characteristic of literature and experimental cancer patient groups.

Validation set	MSI-high	MSI-low/MSS	Total
Colorectal cancer (CRC)			
Current experimental	6	17	23
Current TCGA	85	534	619
Li TCGA	55	320	375
Pacinkova and Popovici TCGA	35	140	175
Danaher TCGA	27	126	153
Endometrial cancer (UCEC)			
Current TCGA	170	363	533
Li TCGA	123	244	367
Pacinkova and Popovici TCGA	52	64	116
Danaher TCGA	71	176	247
Gastric cancer (STAD)			
Current TCGA	71	309	380
Li TCGA	80	335	415
Pacinkova and Popovici TCGA	54	281	335
Danaher TCGA	64	225	289
Uterine carcinosarcoma (UCS)			
Current TCGA	2	53	55
Li TCGA	2	87	89
Pacinkova and Popovici TCGA	—	—	—
Danaher TCGA	—	—	—
Esophageal cancer (ESCA)			
Current TCGA	2	81	83
Li TCGA	2	54	56
Pacinkova and Popovici TCGA	—	—	—
Danaher TCGA	—	—	—
Control			
Current experimental	1	12	13

For the experimental group, we profiled gene expression by RNAseq using FFPE tumor tissue blocks for a total of 23 CRC patients. In addition, we also analyzed a control group of 13 non-CRC tumor blocks to assess MSI signature performance on these samples as well. Among them, five patients had cervical cancer, two had breast cancer, two had gastric cancer, two had glioblastoma, one had ovarian cancer, and one had endometrial carcinosarcoma (Supplementary Table S1). In total, the experimental group (*n* = 36) represented 27 female and nine male patients. The patient age varied from 31 to 84 years; the mean patient age in the experimental group was 60.36 years. More detailed patient information is given in Supplementary Table S1.

We performed RNAseq for each tumor sample and obtained ∼3.75–78.02 million reads uniquely mapped on known human Ensembl genes (genome version GRCh38 and transcriptome annotation GRCh38.89), on the average ∼15.5 million gene-mapped reads per library.

For these samples, “gold standard” MSI statuses were determined by PCR test for seven marker microsatellite loci: BAT25, BAT26, BAT40, NR21, NR24, NR27, and CAT25 that are included in a routinely used clinical panel that requires no healthy tissue control (Suraweera et al., 2002). When there were ≥2 marker loci with detected unstable microsatellite length, these samples were considered MSI-high. Otherwise, the samples were put to the common MSI-low/MSS group. In the experimental group, there were a total of seven MSI-high and 29 MSI-low/MSS samples (Table 1, Supplementary Table S2).

### Performance of Microsatellite Instability RNAseq Gene Signatures

By performing PubMed and Google Scholar literature search with keywords “gene signature,” “gene expression,” “RNA sequencing,” “microsatellite instability,”and “MSI” in March 2021, we extracted 73 hits that were manually processed and returned three recent original publications. These three unrelated research papers authored by Li et al. (2020), Pačínková and Popovici (2019), and Danaher et al. (2019) communicated different gene signatures of MSI status. All these signatures were deduced and initially validated on TCGA CRC samples available at the date of research (Table 1). For all the signatures identified, the initial bioinformatic validation cohorts were smaller than those extracted from TCGA in the current study (Table 1).

The signatures included 15 genes (Li), 25 genes (Pacincova and Popovici), and 14 genes (Danaher) (Figure 1). We compared gene compositions of different signatures and found that they were largely different and shared only one common gene, *MLH1*, which encodes for mutL homolog 1 that can heterodimerize with mismatch repair endonuclease PMS2 to form MutL alpha, part of the DNA mismatch repair system (Figure 1). Li signature shared four other genes with Danaher signature: *EPM2AIP1, RNLS, SMAP1*, and *TTC30A*. These genes encode for EPM2A interacting protein 1, renalase, small ArfGAP 1, and tetratricopeptide repeat domain 30A, respectively. Pacincova and Popovici signature also had two other common genes with Li signature: *RPL22L1* and *SHROOM4* encode for ribosomal protein L22 like 1 and shroom family member 4, respectively. Pacincova and Popovici signature had no other common genes with the Danaher signature (Figure 1).

The experimental and literature samples were then used to assess the performances of those three signatures. All signature values were calculated as described in the original papers. We created and made publicly available the code for signature calculation at Gitlab: https://gitlab.com/ef.viktor/msi_signatures.

The signatures were validated using TCGA RNAseq datasets for tumor samples annotated by MSI status: CRC (*n* = 619), endometrial carcinoma (*n* = 533), gastric cancer (*n* = 380), uterine carcinosarcoma (*n* = 55), and esophageal cancer (*n* = 83) datasets and on the set of experimental CRC RNAseq samples (*n* = 23) and control experimental dataset for non-CRC cancer samples (*n* = 13). To assess signature biomarker quality, we used area under the ROC curve (ROC AUC) value as the measure. AUC reflects biomarker robustness and depends on its sensitivity and specificity (Borisov et al., 2020b). It varies between 0.5 and 1, and the typical discrimination threshold is 0.7, where greater values denote high-quality biomarkers and *vice versa* (Boyd, 1997). AUC is often used for scoring different types of molecular biomarkers in oncology (Liu et al., 2018; Tanioka et al., 2018; Chen et al., 2019; Sorokin et al., 2020a). AUC and 95% confidence intervals were calculated using DeLong’s method implemented in pROC R-package. The entire experimental dataset contained different cancer types; therefore, AUC was calculated only for the CRC subgroup of the experimental samples.

**FIGURE 2:**
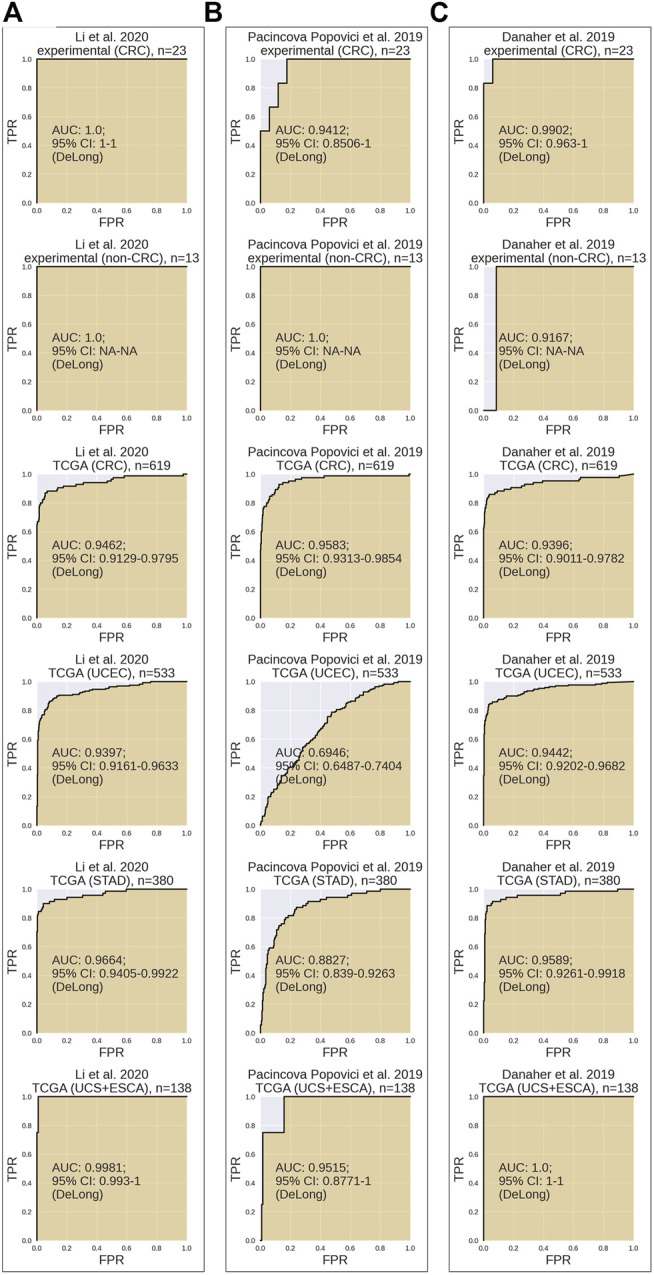
Performance test of MSI RNAseq gene signatures. All signatures were tested for assessment of MSI status on CRC experimental dataset, non-CRC experimental dataset, TCGA CRC dataset, TCGA UCEC dataset, TCGA STAD dataset, and joint TCGA UCS + ESCA dataset. Results for Li et al. (2020) **(A)**, Pačínková and Popovici (2019) **(B)**, and Danaher et al. (2019) **(C)** gene signatures are shown.

In our analysis, *Li* MSI signature (Figure 2A) scored AUC = 1.0 for the experimental CRC dataset, AUC = 0.9462 for the TCGA CRC, AUC = 0.9397 for the TCGA uterine corpus endometrial carcinoma (UCEC), AUC = 0.9664 for the TCGA STAD dataset, and AUC = 0.9981 for the TCGA joint dataset of UCS + ESCA samples. *Pacincova and Popovici* signature (Figure 2B) performed as high as AUC = 0.9412 for the experimental CRC dataset, AUC = 0.9583 for the TCGA CRC dataset, AUC = 0.6946 for the TCGA UCEC, AUC = 0.8827 for the TCGA STAD dataset, and AUC = 0.9515 for the TCGA joint dataset of UCS + ESCA samples. In turn, *Danaher* signature (Figure 2C) showed AUC = 0.9902 for the experimental CRC dataset, AUC = 0.9396 for the TCGA CRC dataset, AUC = 0.9442 for the TCGA UCEC, AUC = 0.9589 for the TCGA STAD dataset, and AUC = 1 for the TCGA joint dataset of UCS + ESCA samples dataset.

**FIGURE 3:**
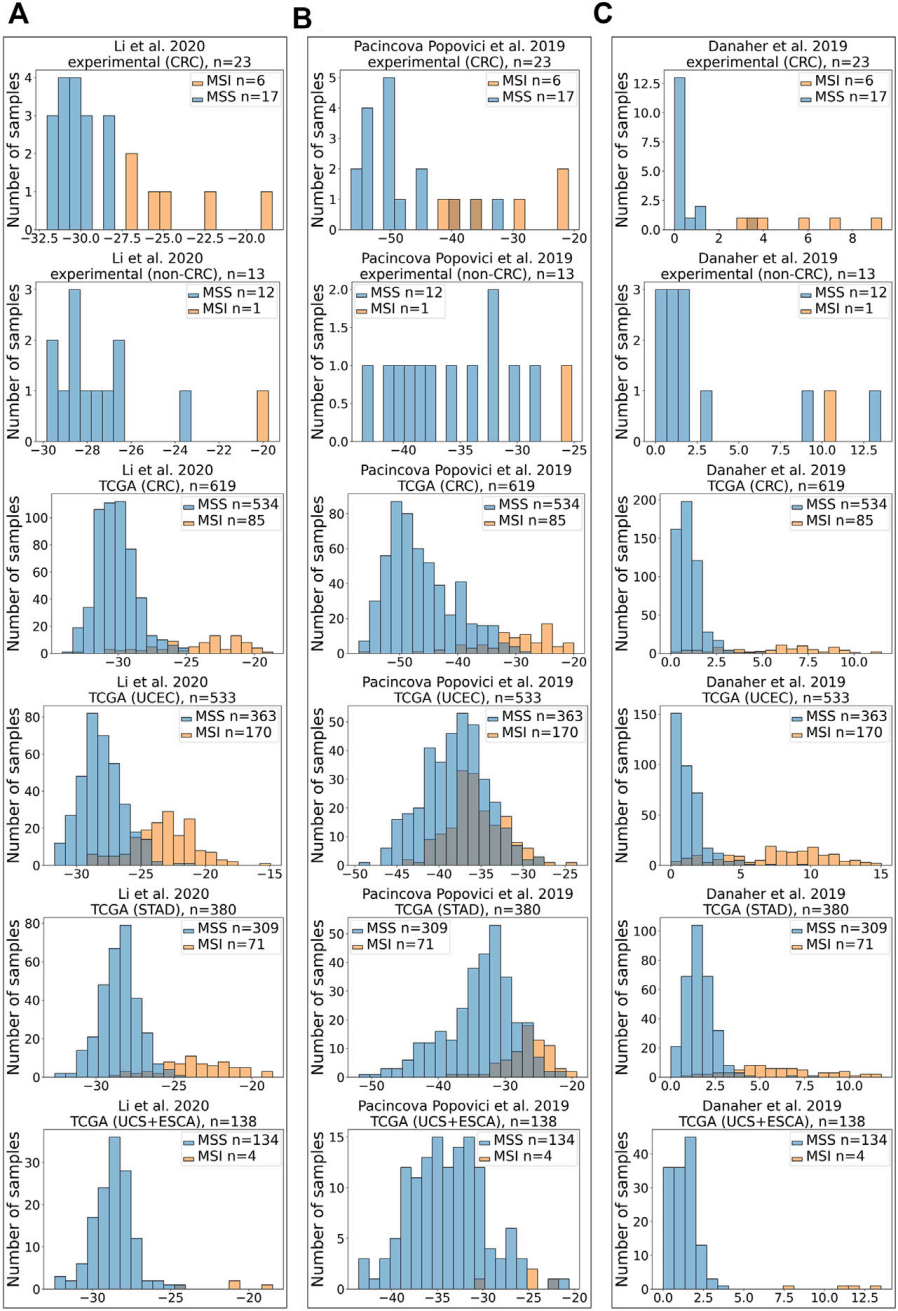
Distribution of scores for MSI RNAseq gene signatures. X-axis shows MSI signature score, Y-axis—number of samples. All signatures were tested for assessment of MSI status on CRC experimental dataset, experimental non-CRC (control) dataset, TCGA CRC dataset, TCGA UCEC dataset, TCGA STAD dataset, and joint TCGA UCS + ESCA dataset. Results for Li et al. (2020) **(A)**, Pačínková and Popovici (2019) **(B)**, and Danaher et al. (2019) **(C)** gene signatures are shown.

Similar to variations in AUC metrics for the three signatures tested, their extents related differently to the true-positive or true-negative MSI statuses (Figures 3A–C).

In the experimental CRC group, there were 6 MSI-high and 17 MSI-low samples. However, in the experimental control group that included non-CRC cancers, there was only one MSI-high sample for endometrial carcinosarcoma, whereas all other samples were MSI-low (Supplementary Table S2). All three signatures supported the true MSI status of samples in the control group (Figures 3A–C).

Assessment of MSI signatures is summarized in Table 2. It can be seen that *Li* signature showed the highest AUC in the experimental CRC group, followed by *Danaher* and *Pacincova and Popovici* signatures, respectively (Table 2). Also, all three signatures performed accurately on TCGA CRC, esophageal cancer, and uterine carcinosarcoma samples with AUC 0.94-1 and highly overlapping 95% confidence intervals. However, in the endometrial carcinoma (UCEC) cohort of TCGA data, *Pacincova and Popovici* signature showed low AUC below 0.7 threshold, whereas two other signatures showed AUC of at least 0.94. The latter also showed lower performance for TCGA gastric cancer samples (AUC = 0.88 *vs*. 0.96–0.97 in the other two signatures).

**TABLE 2:** AUC scores and (95% confidence interval) for three RNAseq MSI gene signatures.

Signature	Li et al. (2020)	Pacinkova and Popovici (2019)	Danaher et al. (2019)
Experimental (CRC), *n* = 23	1.0 (1–1)	0.9412 (0.8506–1)	0.9902 (0.963–1)
TCGA (CRC), *n* = 619	0.9462 (0.9129–0.9795)	0.9583 (0.9313–0.9854)	0.9396 (0.9011–0.9782)
TCGA (UCEC), *n* = 533	0.9397 (0.9161–0.9633)	0.6946 (0.6487–0.7404)	0.9442 (0.9202–0.9682)
TCGA (UCS + ESCA), *n* = 138	0.9981 (0.993–1)	0.9515 (0.8771–1)	1.0 (1–1)
TCGA (STAD), *n* = 380	0.9664 (0.9405–0.9922)	0.8827 (0.839–0.9263)	0.9589 (0.9261–0.9918)

Thus, we conclude that in our tests, all three signatures were equally effective for the CRC, esophageal cancer, and uterine carcinosarcoma samples, whereas for the endometrial carcinomas and gastric cancer samples, the *Danaher* and *Li* signatures were found more effective.

We also separately analyzed only early-stage (stages I, IA, and IB) cancer patients from TCGA. In this case, statistical analysis could be performed only for CRC and gastric cancer groups because there were no early-stage MSI-high patients in the other groups. There were 16/13 MSI-high and 89/42 MSI-low samples in CRC and gastric cancer groups, respectively (Supplementary Figure S1). All three signatures performed accurately on early-stage TCGA CRC with AUC 0.966–0.997 and highly overlapping 95% confidence intervals (Supplementary Figure S2). AUC for *Li* signature was the highest for predicting MSI status in gastric cancer (AUC = 0.956), followed by *Danaher* (AUC = 0.934) and *Pacincova and Popovici* (AUC = 0.919) signatures (Supplementary Figure S2)*.*

## Discussion

In this study, we, for the first time, systematically compared and validated RNAseq gene signatures of MSI status in human solid tumors. All the signatures performed well on both literature and experimental samplings with the MSI statuses determined using the gold standard techniques routinely used in cancer molecular diagnostics. Interestingly, these three signatures were developed by different teams using different logical rationale and were mostly nonoverlapping with only one common gene, *MLH1*, which protein product heterodimerizes to form MutL alpha (Lindner et al., 2021; Pannafino and Alani, 2021), important actor of the DNA mismatch repair system that is widely associated with the Lynch syndrome known as hereditary nonpolyposis CRC, and MSI (Yamamoto and Imai, 2019; Lindner et al., 2021; Stinton et al., 2021).

**FIGURE 4:**
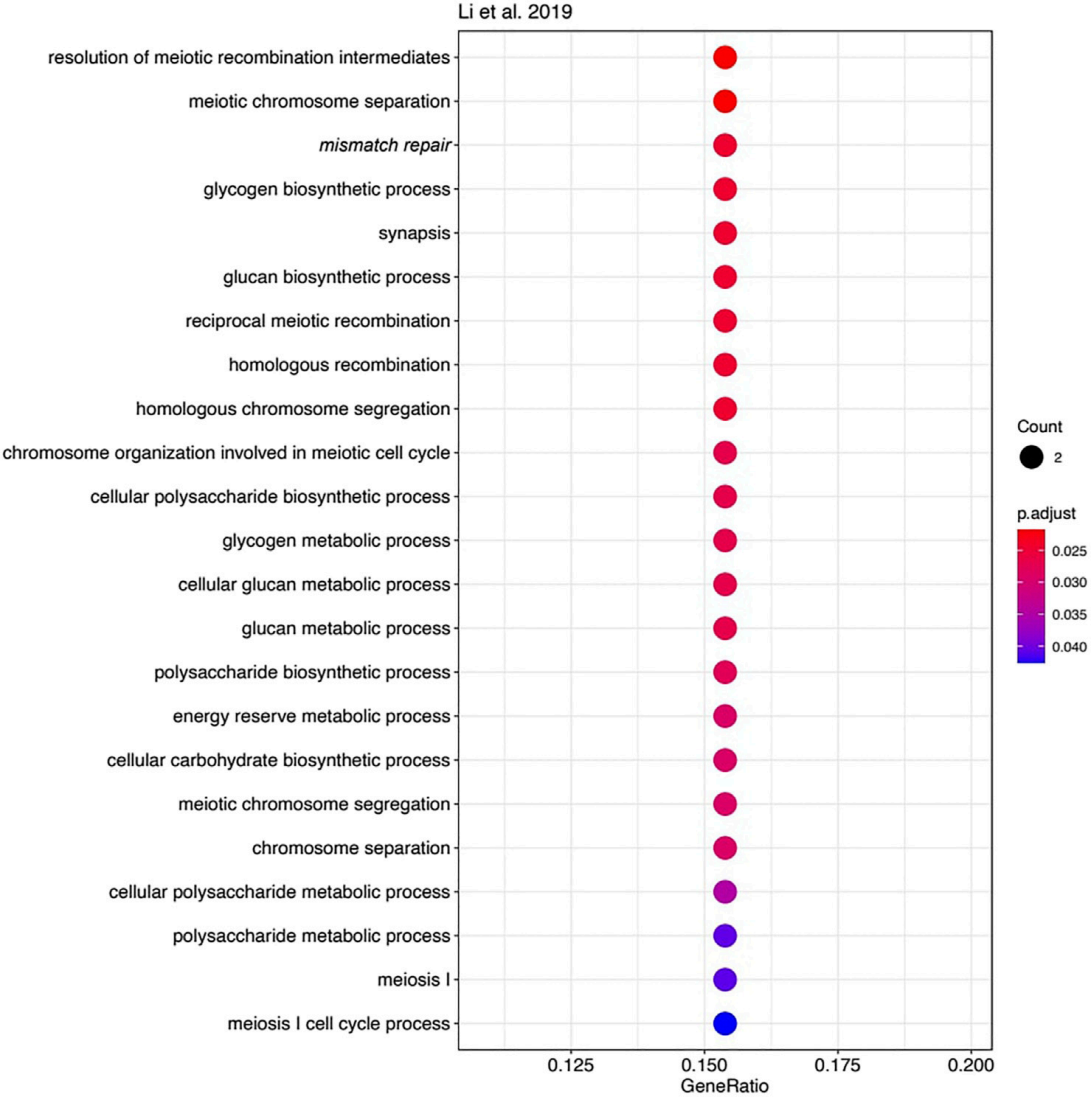
Biological process GO terms for genes included in Li signature. Visualized using R package enrichplot (http://bioconductor.org/packages/release/bioc/html/enrichplot.html). All terms passed Benjamini–Hochberg adjusted *p*-value threshold of 0.05.

**FIGURE 5:**
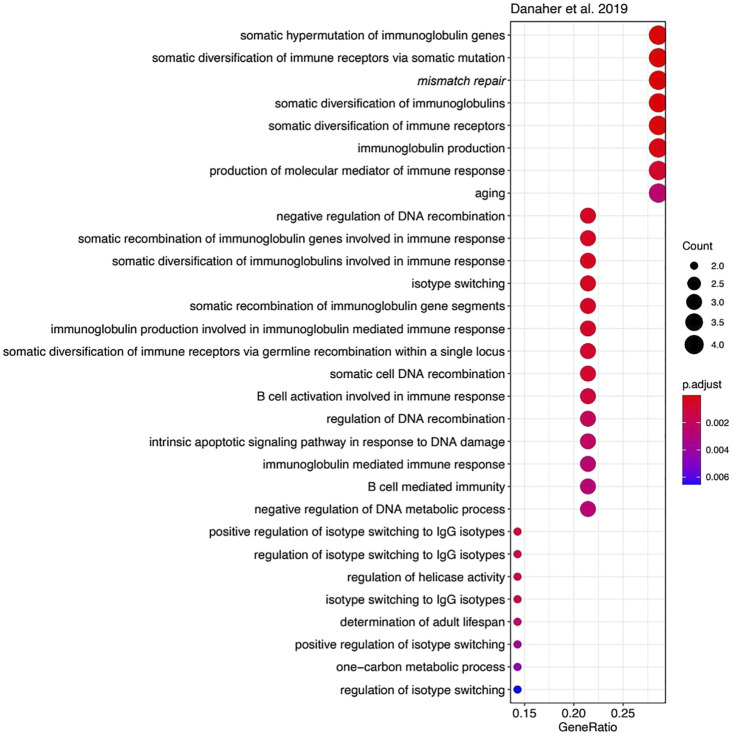
Biological process GO terms for genes included in Danaher signature. Visualized using R package enrichplot (http://bioconductor.org/packages/release/bioc/html/enrichplot.html). All terms passed Benjamini–Hochberg adjusted *p*-value threshold of 0.05.

However, the functions of most other genes in the three MSI signatures strongly differ. We used Gene Ontology (GO) analysis to identify GO term “biological processes” enriched among the genes forming each signature. Of note, we found 23 enriched biological processes in *Li* gene signature (Figure 4), 30 in *Danaher* signature (Figure 5), and no significantly enriched processes in *Pacincova and Popovici* signature.

The most significant terms in *Li* signature were associated with meiosis, mismatch repair, and (unexpectedly) with glycogen biosynthesis (Figure 4). Interestingly, there were previously only indirect links reported for the glycogen metabolism and Lynch syndrome (Kato, 2020) or MSI (Krausova and Korinek, 2014; Oh et al., 2016), e.g., through the Wnt signaling pathway (Krausova and Korinek, 2014). In *Danaher* signature, the most significant terms were associated with mismatch repair and with somatic hypermutation of immunoglobulin genes and physiologically related processes: somatic diversification of immune receptors and immunoglobulins (Figure 5). The latter feature is widely associated with Lynch syndrome and MSI (Anghileri et al., 2021; Mäki-Nevala et al., 2021). Among the signatures by *Li* and *Danaher*, “Mismatch repair” was the only common GO term (highlighted in italic on Figures 4 and 5), and mismatch repair deficiency is one of the most obvious reasons for MSI (Jin and Sinicrope, 2021). However, analysis of *Pacincova and Popovici* signature returned no enriched functional terms, thus evidencing that it contains quite a functionally heterogeneous gene set.

We then performed Kyoto Encyclopedia of Genes and Genomes (KEGG) pathway enrichment and gene set enrichment (GSEA) analyses. The analyses returned three common statistically significantly enriched pathways for *Danaher* signature: “Mismatch repair,” “Platinum drug resistance,” and “Colorectal cancer” (Supplementary Figures S3 and S4). Thus, GSEA and KEGG analyses confirmed our previous finding that *Danaher* signature is enriched by mismatch repair genes. However, neither KEGG pathway enrichment nor GSEA provided significantly enriched pathways for both *Pacincova and Popovici* and *Li* signatures.

This apparent gene content diversity among the signatures demonstrates that MSI can be associated with several or many processes that are not exclusively linked with DNA hypermutation and repair. This gives hope for building next-generation MSI signatures with even better performance/classifier scores.

Our results also imply that the *Li* and *Danaher* signatures may be effective for the CRCs, esophageal cancers, uterine carcinosarcomas, endometrial carcinomas, and gastric cancers. However, the overall effectiveness of *Pacincova and Popovici* signature in our tests was lower and limited to the first three among the cancer types discussed earlier. Moreover, all three signatures performed well for predicting MSI status in early-stage CRC and gastric cancer. Interestingly, the *Li* and *Danaher* signatures that were significantly enriched by genes for certain biological processes (Figures 3 and 4) were effective for more cancer types than *Pacincova and Popovici* signature that lacked enriched GO terms.

In addition, the current experimental dataset may serve for validating new such signatures. Finally, we implemented here all the MSI signatures assessed as the free code ready to use with the user RNAseq data. In the future and after careful clinical validation, this may have a practical significance for establishing MSI statuses by screening, when available, RNAseq data for the cancers not necessarily strongly associated with the Lynch syndrome.

## Materials and Methods

### Patients and Samples

In this study, we investigated MSI status-annotated RNAseq profiles for a total of 1,693 cancer samples (one sample per individual patient). Among them, there were 619 literature CRC samples from TCGA cohort, 533 TCGA UCEC samples, 380 TCGA gastric cancer samples, 55 TCGA uterine carcinosarcoma samples, 83 TCGA esophageal cancer samples, and 36 experimental samples profiled by RNA sequencing in this study. TCGA RNAseq samples were extracted from five source datasets: COAD (colon cancer, *n* = 389) and READ (rectal cancer, *n* = 230) for “CRC,” UCEC (endometrial carcinoma, *n* = 533), STAD (gastric cancer, *n* = 380), UCS (uterine carcinosarcoma, *n* = 55), and ESCA (esophageal cancer, *n* = 83). MSI annotated TCGA data were downloaded from https://gdac.broadinstitute.org/.

The experimental dataset included 23 colon cancer samples, five cervical cancer samples, two breast cancer, two gastric cancer samples, two glioblastoma samples, one ovarian cancer sample, and one endometrial carcinosarcoma sample. All experimental specimens were stored in the form of FFPE tissue blocks.

### Gene Expression Profiling

To isolate RNA, 10-µM thick paraffin slices were trimmed from each FFPE tissue block using a microtome. RNA preps were extracted using QIAGEN RNeasy FFPE Kit. RNA 6000 Nano or Qubit RNA Assay kits were used to measure RNA concentration. RNA integrity number was measured using Agilent 2100 bio-Analyzer. For depletion of ribosomal RNA and library construction, KAPA RNA Hyper with rRNA erase kit (HMR only) was used. Different adaptors were used for multiplexing samples in one sequencing run. Library concentrations and quality were measured using Qubit ds DNA HS Assay kit (Life Technologies) and Agilent Tapestation (Agilent). RNA sequencing was done using Illumina NextSeq 550 equipment for single-end sequencing, 50-bp read length, for approximately 30 million (mln) raw reads per sample. Data quality check was done on Illumina SAV. De-multiplexing was performed with the Illumina Bcl2fastq2 v 2.17 program. Sequencing data were deposited in National Center for Biotechnology Information Sequencing Read Archive under accession ID PRJNA744404.

### Processing of Experimental RNAseq Data

RNAseq FASTQ files were processed with STAR aligner (Dobin et al., 2013) in “GeneCounts” mode with the Ensembl human transcriptome annotation (Build version GRCh38 and transcript annotation GRCh38.89). Ensembl gene IDs were converted to HUGO Gene Nomenclature Committee (HGNC) gene symbols using the Complete HGNC dataset (https://www.genenames.org/, database version from July 13, 2017). Totally, expression levels were established for 36,596 annotated genes with the corresponding HGNC identifiers. Quantile normalization (qnorm python package) was used to normalize gene expression values.

### Calculating Li et al. Signature Values

MSI RNAseq signature described by Li et al. (2020) was calculated according to the original paper. This signature defines *LYG1*, *MSH4*, and *RPL22L1* genes as “plus”-genes and *DDX27, EPM2AIP1, HENMT1, MLH1, NHLRC1, NOL4L, RNLS, RTFDC1, SHROOM4, SMAP1, TTC30A,* and *ZSWIM3* as “minus”-genes. The final score is a sum of log10-transformed normalized gene expression levels with consideration of each gene sign.

### Calculating Pacincova and Popovici Signature

MSI RNAseq signature described by Pacincova and Popovici was calculated according to the original paper (Pačínková and Popovici, 2019). This signature defines *AGR2*, *TNNT1*, *VNN2*, *TNFSF9*, *TRIM7*, and *RPL22L1* genes as “plus”-genes and *ACSL6*, *ARID3A*, *ASCL2*, *AXIN2*, *EPDR1*, *GGT7*, *GNG4*, *KHDRBS3*, *KRT23*, *MLH1*, *NKD1*, *PLAGL2*, *PRR15*, *RUBCNL*, *SHROOM2*, *SHROOM4*, *TFCP2L1*, *TNNC2*, and *VAV3* genes as “minus”-genes. The final score is a sum of log10-transformed gene expression levels with consideration of each gene sign.

### Calculating Danaher et al. Signature

MSI RNAseq signature described by Danaher et al. (2019)was calculated according to the original paper. This signature includes *MLH1, MSH2, MSH6,* and *PMS2* genes for calculating MMR loss score (MLS). First, a minimal Z-score (Zmin) of log2-transformed gene expressions was found. The final MLS = (Zmin + 1.03)/0.69, where 1.03 and 0.69 are the theoretical expectation and standard deviation of the minimum of four standard normal random variables, respectively.

Hypermutation predictor score was calculated by multiplying log2-transformed expressions of *EPM2AIP1, TTC30A, SMAP1, RNLS, WNT11, SFXN1, SREBF1, TYMS, EIF5AL1,* and *WDR76* genes by coefficients from the table given in the original article. The final hypermutation predictor score is a Z-score of the calculated value. The resulting MSI predictor score was calculated as follows:

$$\sqrt{\min{\left(\mathrm{MLS},0\right)}^{2}+\max{\left(\mathrm{HPS},0\right)}^{2}}$$

The MSI predictor score is further used as a predictor of MSI-high status.

### Functional Gene Set Enrichment Analysis

KEGG and GO analyses were performed using the R clusterProfiler package. EnrichKEGG and enrichGO functions were used to implement enrichment analysis. GSEA analysis was performed using the web service http://www.webgestalt.org. The following non-default parameters were selected: KEGG pathways were used as a functional database, and the minimum number of genes for a category was set to 3. We used Benjamini–Hochberg false discovery rate correction method and applied a *p*-value threshold of 0.05 as a cutoff value for filtering pathways and GO terms.

### Experimental Microsatellite Instability Assessment by Polymerase Chain Reaction

Genomic DNA was isolated from FFPE tissue sections using the QIAamp DNA FFPE Tissue Kit (Qiagen, Valencia, CA).

We performed MSI analysis using a set of five so-called “main” mononucleotide repeat markers: *BAT25*, *BAT26*, *NR21*, and *NR24* selected from the revised Bethesda panel (Suraweera et al., 2002) and *NR27* selected from the modified pentaplex panel (Buhard et al., 2006). Two additional mononucleotide repeat markers were also included: *BAT40*, as it was shown to improve the sensitivity of MSI testing in both CRC and extra-colonic tumors (Hartmann et al., 2002; Pagin et al., 2013) and *CAT-25*, which was reported to increase the sensitivity for identifying d*MSH6* tumors (Takehara et al., 2018).

The primer sequences were taken from previous reports (Hartmann et al., 2002; Suraweera et al., 2002; Buhard et al., 2006; Takehara et al., 2018). The sequences of fluorescently labeled oligonucleotides are listed in Table 3.

**TABLE 3:** Oligonucleotide sequences and fluorescent labels used.

Marker	Gene		Primer sequence and fluorescent labels (5′-3′)	Length (bp)
BAT26	*hMSH2*	Forward	FAM-CTGCGGTAATCAAGTTTTTAG	183
		Reverse	AAC​CAT​TCA​ACA​TTT​TTA​ACC​C	
BAT25	*c-kit*	Forward	R6G-TACCAGGTGGCAAAGGGCA	153
		Reverse	TCT​GCA​TTT​TAA​CTA​TGG​CTC	
NR24	*Zinc finger 2 (ZNF-2)*	Forward	TAMRA-GCTGAATTTTACCTCCTGAC	131
		Reverse	ATT​GTG​CCA​TTG​CAT​TCC​AA	
NR21	*SLC7A8*	Forward	FAM-GAGTCGCTGGCACAGTTCTA	109
		Reverse	CTG​GTC​ACT​CGC​GTT​TAC​AA	
NR27	*Inhibitor of apoptosis Protein-1 (IAP1)*	Forward	R6G-AACCATGCTTGCAAACCACT	87
		Reverse	CGA​TAA​TAC​TAG​CAA​TGA​CC	
BAT40	*3-β-hydroxysteroid dehydrogenase (HSD3B1)*	Forward	ROX-AGTCCATTTTATATCCTCAAGC	145
		Reverse	GTA​GAG​CAA​GAC​CAC​CTT​G	
CAT25	*Caspase 2*	Forward	ROX-CTTCCCAACTTCCCTGTTCTTT	109
		Reverse	TGA​GCT​GAG​ATC​GTG​CCA​CT	

The marker DNA products were PCR amplified using the qPCRmix-HS (Evrogen, Russia). PCR was carried out in a 20-μl final volume containing 1× qPCRmix-HS, 2 *p*moles of each primer, and approximately 20 ng of DNA template.

The marker sets (1) *BAT25*, *BAT26*, *NR21*, and *NR27* and (2) *BAT-40* and *CAT-25* were co-amplified in one PCR tube per set. The marker *NR-24* was amplified in a separate PCR tube.

PCR conditions for the tetraplex and duplex assays consisted of an initial 2-min denaturation step at 94C, followed by 37 cycles at 94°C for 20 s, 54°C for 10 s, and 72°C for 12 s, with a final extension at 72°C for 2 min. Conditions for monoplex reaction differed in annealing temperature: 53°C.

Amplified PCR products were analyzed by capillary electrophoresis performed on ABI prism 3130 × l System (Applied Biosystems, United States). The microsatellite marker lengths were detected by Sequence Scanner software (Applied Biosystems, United States).

The cutoff for MSI status classification was chosen on the basis of the threshold of approximately 40%, according to Umar A. et al. (2004). Tumors with instability at ⩾2 of the five main mononucleotide markers were defined as MSI-H. Samples with instability at one main marker were further tested with the additional markers. Tumors with at least one unstable additional marker were defined as MSI-high. Otherwise, tumors were classified as MSI-low/MSS.

## Data Availability

The datasets presented in this study can be found in online repositories. The names of the repository/repositories and accession number(s) can be found below: https://www.ncbi.nlm.nih.gov/bioproject/PRJNA744404.
