# Supplementary figures and images for: Experimental and Meta-Analytic Validation of RNA Sequencing Signatures for Predicting Status of Microsatellite Instability
Source: Front Mol Biosci. 2021 Nov 23;8:737821. doi: 10.3389/fmolb.2021.737821 (PMC8650122; doi:10.3389/fmolb.2021.737821)

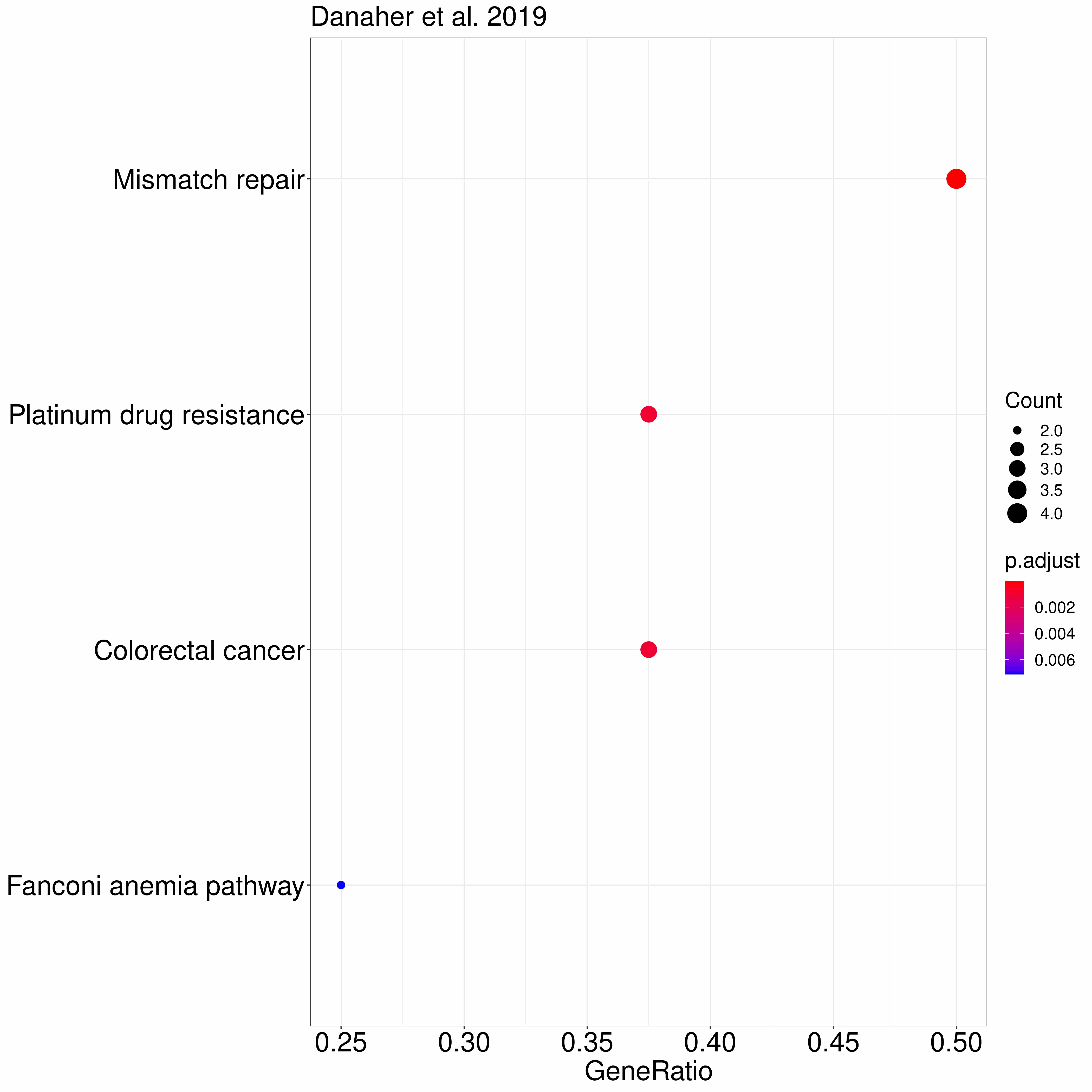

Supplement: Supplementary file 1 [file Image4.PNG]

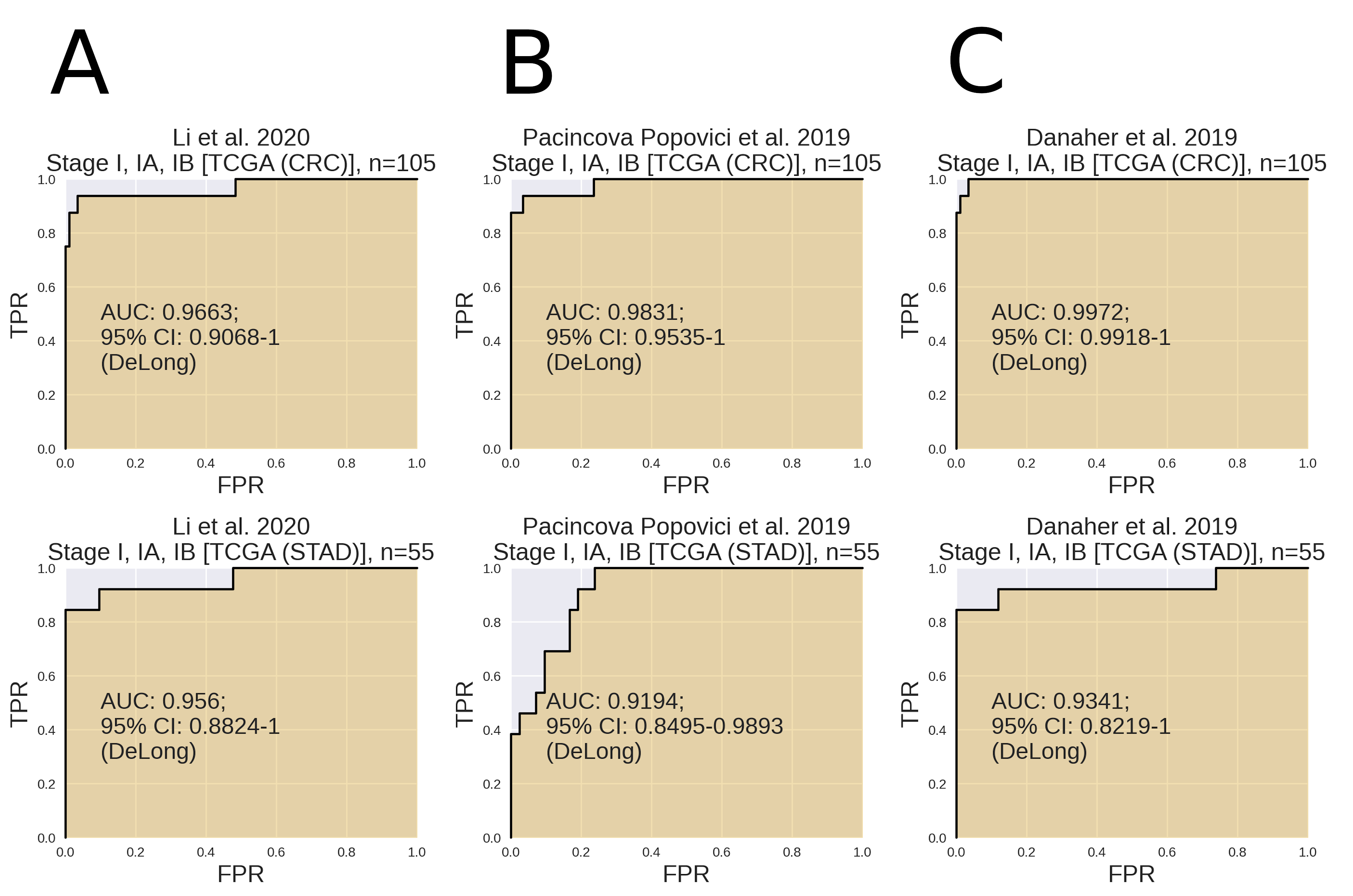

Supplement: Supplementary file 3 [file Image2.PNG]

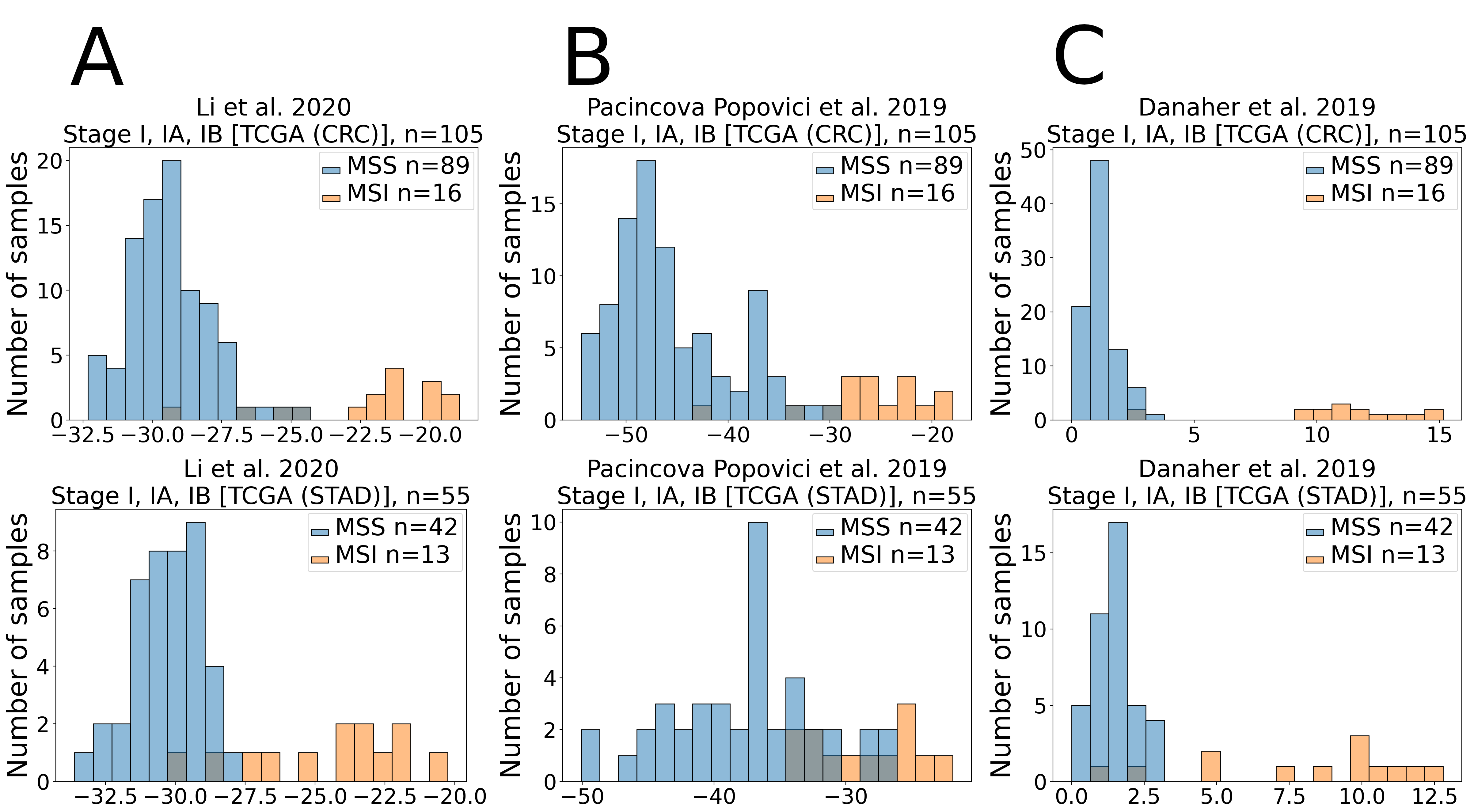

Supplement: Supplementary file 5 [file Image1.PNG]

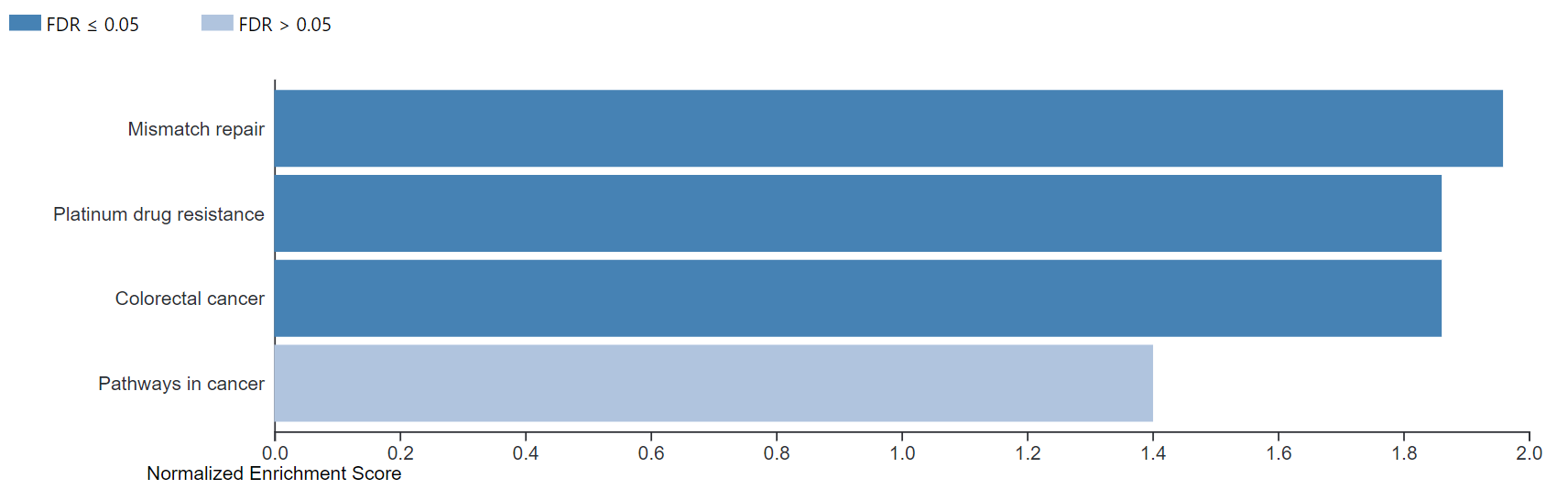

Supplement: Supplementary file 6 [file Image3.PNG]
