# Supplementary material for: Experimental and Meta-Analytic Validation of RNA Sequencing Signatures for Predicting Status of Microsatellite Instability
Source: Front Mol Biosci. 2021 Nov 23;8:737821. doi: 10.3389/fmolb.2021.737821 (PMC8650122; doi:10.3389/fmolb.2021.737821)
Supplement: Supplementary file 2 [file Table2.DOCX]

**Table S2. Cancer type content of the experimental dataset**

| **Cancer type** | **MSI-high** | **MSI-low/MSS** | **Total** |
| --- | --- | --- | --- |
| Colorectal cancer group | | | |
| Colorectal cancer | 6 | 17 | 23 |
| Total, CRC group | 6 | 17 | 23 |
| Control group | | | |
| Cervical cancer | 0 | 5 | 5 |
| Breast cancer | 0 | 2 | 2 |
| Gastric cancer | 0 | 2 | 2 |
| Glioblastoma | 0 | 2 | 2 |
| Ovarian cancer | 0 | 1 | 1 |
| Endometrial carcinosarcoma | 1 | 0 | 1 |
| Total, control group | 1 | 12 | 13 |
